# Quantifying Diet Intake and Its Association with Cardiometabolic Risk in the UK Airwave Health Monitoring Study: A Data-Driven Approach

**DOI:** 10.3390/nu12041170

**Published:** 2020-04-22

**Authors:** Larissa C. Hunt, Hassan S. Dashti, Queenie Chan, Rachel Gibson, Céline Vetter

**Affiliations:** 1 Department of Integrative Physiology, University of Colorado Boulder, Boulder, CO 80309, USA; larissa.hunt@colorado.edu; 2 Center for Genomic Medicine, Massachusetts General Hospital and Harvard Medical School, Boston, MA 02114, USA; hassan.dashti@mgh.harvard.edu; 3 Department of Epidemiology and Biostatistics, School of Public Health, Imperial College London, London W2 1PG, UK; q.chan@imperial.ac.uk; 4 Department of Nutritional Sciences, School of Life Course Sciences, King’s College London, London SE1 9NH, UK

**Keywords:** nutrient intake, diet patterns, food intake regularity, meal frequency, cluster, body mass index, Airwave Health Monitoring Study

## Abstract

We used data-driven approaches to identify independent diet exposures among 45 candidate variables, for which we then probed cross-sectional associations with cardiometabolic risk (CMR). We derived average daily caloric intake and macronutrient composition, daily meal frequencies, and irregularity of energy and macronutrient intake from 7-day food diaries in the Airwave Health Monitoring Study participants (*N* = 8090). We used K-means and hierarchical clustering to identify non-redundant diet exposures with representative exposures for each cluster chosen by silhouette value. We then used multi-variable adjusted logistic regression to estimate prevalence ratios (PR) and 95% confidence intervals (95%CI) for CMR (≥3 criteria: dyslipidemia, hypertension, central adiposity, inflammation and impaired glucose control) across diet exposure quartiles. We identified four clusters: i) fat intake, ii) carbohydrate intake, iii) protein intake and intake regularity, and iv) meal frequencies and energy intake. Of these clusters, higher carbohydrate intake was associated with lower likelihood of CMR (PR = 0.89, 95%CI = 0.81–0.98; *p_trend_* = 0.02), as was higher fiber intake (PR = 0.76, 95%CI = 0.68–0.85; *p_tren_*_d_ < 0.001). Higher meal frequency was also associated with lower likelihood of CMR (PR = 0.76, 95%CI = 0.68–0.85; *p_trend_* < 0.001). Our results highlight a novel, data-driven approach to select non-redundant, minimally collinear, primary exposures across a host of potentially relevant exposures (including diet composition, temporal distribution, and regularity), as often encountered in nutritional epidemiology.

## 1. Introduction

Obesity and associated chronic cardiometabolic health conditions are major public health concerns. In the UK, 26% of adults are classified as obese [1] and nearly a quarter of all deaths are caused by cardiovascular diseases [2]. In the US, cardiovascular disease is the leading cause of death and its prevalence is expected to increase to 45% of adults in the next 15 years [3]. Diet is an established modifiable risk factor for chronic diseases. Decades of nutrition research have identified numerous dietary factors as relevant to human health, and numerous clinical trials have targeted such factors with the goal of preventing and treating chronic diseases, including obesity, metabolic disorders, and cardiovascular disease.

Nutrition research has traditionally focused on single nutrients, overall diet quality, and/or composition and their associations on cardiometabolic health. For example, Key et al. [4] examined the risk of ischemic heart disease associated with a range protein sources, including eggs, meat, dairy, and fish in more than 400,000 individuals participating in the European Prospective Investigation into Cancer and Nutrition (EPIC) cohort study. They observed an increased risk of heart disease for individuals reporting higher processed and red meat consumption, but not fish, poultry, or milk. Another prospective UK cohort study examined breakfast energy intake (estimated from a 7-day food diary) and showed that individuals with the highest breakfast energy intake levels had the lowest body mass index (BMI), despite higher overall energy intake, and gained less weight over a two-year follow-up period [5]. During the last decade, nutritional epidemiology has also moved towards the study of dietary patterns and combinations of different nutrients. For example, in a prospective analysis of 75,020 women in the Nurses’ Health Study and 42,865 men of the Health Professionals Follow-Up Study, AlEssa et al. [6] found that higher carbohydrate to cereal fiber ratio as well as higher starch to cereal fiber ratio are associated with an higher risk of coronary heart disease. Their findings highlight the importance of studying nutrient intakes in tandem as these may provide more information on the quality of food consumed.

More recently, timing, frequency, and regularity of food intake have emerged as novel risk factors for cardiometabolic health [7,8,9,10,11]. For example, Ma et al. [12] conducted a cross-sectional analysis in 499 participants within the SEASONS (Seasonal Variation of Blood Cholesterol) Study using 24-h dietary recalls and body weight measurements. Their results indicated that those individuals reporting ≥ 4 eating occasions per day had a 45% lower risk of obesity compared to individuals that ate less frequently (≤ 3 times per day, OR 0.55, 95%CI = 0.33–0.91), independent of daily energy intake. Pot et al. [13] have proposed a novel method of assessing the inconsistency of day to day energy intake. In their study of 1768 individuals who completed 5-day food diaries, they quantified energy intake during predefined meals (breakfast, lunch, and dinner), between meal intake, and daily intakes compared to a 5-day mean energy intake. The results showed that higher irregularity scores of energy intake, particularly for breakfast and between meal intake, were associated with an increased risk of metabolic syndrome (OR 1.34, 95% CI = 0.99–1.81 and OR 1.36, 95% CI = 1.01–1.85, respectively). The findings of this study provide compelling evidence to consider regularity of energy intake when examining eating patterns. However, to this date it is unclear how regularity of additional nutrients (proteins, carbohydrates, and fats) may affect cardiometabolic health and how regularity may interact with the other dimensions of eating patterns.

From this body of evidence, individual dietary intake dimensions (composition, frequency, regularity) are novel predictors of cardiometabolic disease risk, but the concurrent study of multiple of these dimensions is so far under-developed. Understanding diet exposures that need to be considered in relation to cardiometabolic disease could provide critical insights to novel design strategies for targeted dietary interventions to address the global obesity and cardiovascular disease pandemics. It could also help address the challenge of multiple comparisons, which has been raised in the past [14,15]. The goal of this study was to undertake a data-driven approach to identify non-redundant, minimally collinear diet exposures from 7-day food diaries, which we then used to probe associations with cardiometabolic risk (CMR) in the Airwave Health Monitoring Study (AHMS). This exploratory, proof-of-principle study was designed to evaluate the potential of data-driven methods to address challenges that arise when modeling large numbers of potentially highly correlated measures.

## 2. Materials and Methods

### 2.1. Sample and Study Design

The AHMS is an occupational cohort launched in June 2004 enrolling police personnel across Great Britain and a total of 53,114 participants were enrolled by end of recruitment in March 2015. All members of the police force in Great Britain (including England, Scotland, Wales, and Northern Ireland) were eligible for enrollment, and recruitment and baseline measurements have been described previously [16]. For the purposes of our study, we included AHMS participants with baseline dietary data available by December 2012 (*N* = 9018). The AHMS is approved by the National Health Service Multi-Site Research Ethics Committee (MREC/13/NW/0588); each participant provided informed written consent.

### 2.2. Dietary Assessment

Participants completed 7-day food diaries with 8 predefined eating occasions (before breakfast, breakfast, mid-morning, lunch, tea, evening meal, later evening, and anything else not covered) for intake reporting. Participants were instructed to provide information on cooking methods, brand names, and serving sizes for each report. Participants were also provided pictures to better help them estimate serving size based on those developed by Nelson et al. [17]. Nutrition intake was then calculated using Dietplan6.7 software (Forestfield Software Ltd., Horsham, UK), which was based on the McCance and Widdowson’s 6th Edition Composition of Foods UK Nutritional Dataset (UKN). Trained coders (i.e., dieticians, nutritionists, or personnel working towards nutrition qualifications) matched food and drink reports to the UKN database code and portion size [18]. Participants recording less than 500 kilocalorie (kcal) per day, which is considered to be physiologically unsustainable, were excluded by dietary coders from the sample [18].

### 2.3. Diet Exposure Quantification

We derived 45 variables to represent the diet exposures known to influence cardiometabolic health (a full list of derived variables can be found in Table S1). Briefly, we derived average daily energy intake and energy-adjusted intake (percent contribution to daily energy intake) of proteins, carbohydrates, sugar, fats and saturated fats, as well as fiber intake as g/1000 kcal to represent the components of food intake. We also derived meal-specific average energy intake for breakfast, lunch, dinner and snacks, and meal-specific energy-adjusted intake of protein, carbohydrates, sugar, fat and saturated fats, as well as fiber intake (g/1000 kcal). Snacks comprised of energy intake remaining after subtracting meal-specific energy intake from daily energy intake.

In order to account for meal skipping and distinct temporal patterns of diet intake, we also derived frequency of breakfast, lunch, dinner, late-night snacks, as well as the frequency of having a morning and a late-night snack together. These frequencies were then expressed as percentages of how many times a participant recorded the consumption of that eating occasion out of all recorded days. We defined every intake ≥50 kcal as an eating occasion [19].

Finally, we calculated irregularity scores out of 100 (with a score of zero being the most regular and a score of 100 being the most irregular) for daily energy intake, protein, carbohydrate, sugar, fat, saturated fats and fiber intake, as well as meal-specific energy intake following the algorithms described by Pot et al. [13], but adapted to a 7-day instead of a 5-day food diary recording period. The irregularity score is calculated as the absolute difference of intake on each individual day from the mean intake across all valid days of recording. This is then divided by the mean intake across all valid days of recording, multiplied by 100, and then averaged over the valid days of recording. All derivations were conducted using R Studio version 3.5.3 [20] and variables were log-transformed and z-scaled in order to normalize distributions.

### 2.4. Anthropometric, Blood Pressure and Biochemical Measurements

Participants enrolled in the AHMS attended health-screening visits at a regional clinic. Standard protocol conducted by trained research nurses has been described previously [16]. Participant body weight was measured to the nearest 0.05 kg using digital scales (Marsden digital weighing scale), and height to the nearest 0.1 cm (Marsden H226 portable stadiometer). BMI was calculated as height (kg)/weight (m^2^). Waist circumference was measured between the lower rib margin and iliac crest in the mid-axillary line using a Wessex-finger/joint measure tape (Seca 201, Seca, Birmingham UK). Blood pressure measurements were made from the seated position using the Omron HEM 705-CP digital BP monitor (Omron Health Care). An average of three separate measurements taken 30 s apart was recorded. Non-fasted state venous blood samples were collected and processed on site, then transported (stored in a thermoporter at 0–4 °C) to be processed further at a designated study laboratory. Serum was used for high density lipoprotein (HDL), non-HDL, cholesterol, high sensitivity-C-reactive protein (Hs-CRP) assays; whereas whole EDTA blood samples (IL 650 analyzer Instrumentation Laboratory, Bedford, Massachusetts, USA) were used for glycated hemoglobin tests (HbA1c).

### 2.5. Outcome Definition: Cardiometabolic Risk

We defined CMR in line with prior work in the AHMS [21], requiring for each participant to have three or more of the following factors to be qualified as a case, with controls being those participants with fewer than three of the factors:

1. Central adiposity (waist circumference ≥ 94 cm [men] and ≥ 80 cm [women]),
2. Dyslipidemia (HDL <1.0 mmol/L [men] and <1.3 mmol/L [women], and/or non-HDL ≥ 4.0 mmol/L, and/or prescribed lipid lowering medication),
3. Elevated blood pressure (systolic ≥ 130 mmHg, and/or diastolic ≥ 85 mmHg, and/or prescribed hypotensive medication),
4. Inflammation (Hs-CRP ≥ 3 mg/L < 10 mg/L),
5. Impaired blood glucose control: HbA1c ≥ 5.7% and/or prescribed medication for glucose control)

### 2.6. Covariate Assessment

Participant occupational, lifestyle, medical history, sociodemographic, and demographic information was collected during the health-screening visit using an on-line questionnaire. Participant educational attainment level (A levels/Higher or equivalent, Bachelor’s degree or higher, and other) and participant relationship status (single, married/cohabiting, and divorced/separated) were collapsed into three categories. Participant annual household income was categorized into one of the following categories: less than £37,999, £37,999–77,999, and more than £78,000. Total working hours (including usual weekly overtime) was classified into two categories (> 40 hours and ≥ 40 hours per week). Physical activity information was collected with the International Physical Activity Questionnaire Short Form (IPAQ-SF) [22]. Metabolic equivalent (MET) minutes per week across three exercise categories (walking, moderate, and vigorous) are calculated and participants are categorized by high, moderate, or low levels of activity [23]. Finally, self-reported sleep duration was categorized as less than 7 hours, 7–8 hours, or 9 hours or more.

### 2.7. Statistical Methods

We used variable dimension reduction techniques to identify relevant (i.e., non-redundant and minimally collinear) diet exposures for statistical modeling, and then implemented logistic regression models to examine associations with CMR. For the purposes of the present analyses, we excluded participants if they had missing diet variable information (*N* = 150) and further excluded participants (*N* = 277) with fewer than 5 valid days of recording, defining a valid day with a 500-kcal cutoff. We also excluded participants with self-reported chronic disease diagnosis, including angina, thyroid disease, heart disease, chronic obstructive pulmonary disease, cancer, chronic liver disease, and previous stroke (*N* = 499), as reported in previous analyses [21] as these chronic diseases may cause changes in eating habits. We also excluded participants with incomplete outcome information (*N* = 2) for a final analytical sample of 8090 participants. All statistical analyses were conducted using R Studio version 3.5.3 [20].

#### 2.7.1. Dimension Reduction Techniques and Diet Exposure Selection

Unlike the typical use of k-means clustering in prior analysis, where the goal is to cluster participants with similar dietary intake patterns [24,25,26,27], our goal was to identify how variables cluster together, so that we could identify independent diet exposures, and use this information for follow-up modeling analysis of CMR. Our dimension reduction technique was k-means clustering and for primary analyses we determined the optimal number of clusters to use for k-means by using the Bayesian Information Criterion (BIC) (ClusterR package version 1.1.9 [28] and cluster package version 2.0.7–1 [29]). Using this criterion, a lowest BIC indicates best model fit and thus the optimal number of clusters.

It is noteworthy that there are many methods and indices to determine the ideal numbers of clusters, and as often the case for exploratory machine learning algorithms, there is no single best one. We therefore also used the NbClust package version 3.0 [30] to obtain a distribution of ideal cluster numbers using 21 alternative criteria to the BIC, which could further inform and guide secondary analyses. We also implemented hierarchical clustering of the variable set to identify potential alternative, secondary clusters (corrplot package version 0.84 [31]). Results of hierarchical clustering, presented as a dendrogram (tree diagram) of diet exposure relationships, together with the results from the NbClust package [30], were used to inform the optimal number of clusters to use for secondary k-means clustering analyses, where potentially divergent clusters would be broken up into smaller, more cohesive clusters.

Using this k-means clustering approach, each diet exposure is uniquely assigned to a given cluster, based on their distance to the assigned cluster’s centroid and the neighboring cluster centroids; this metric is typically referred to as a silhouette value. Silhouette values range from +1 to −1, with a value of +1 indicating the variable is located directly on top of its cluster centroid and thus its best representation. We chose the variable with the highest silhouette value as the main exposure for the subsequent analyses.

Finally, to evaluate potential collinearity of the identified exposure variables, we correlated the exposure variables identified by k-means analyses. In order to minimize the case that the correlation between the two top variables of a given cluster was <0.50, we also included the second hit variable for a given cluster in subsequent regression analyses.

#### 2.7.2. Examination of Association Between Identified Diet Exposures and Cardiometabolic Risk

We used logistic regression models to examine the cross-sectional associations of the relevant diet exposures—determined during the dimension reduction step—with CMR. Due to the elevated prevalence of CMR (>10%) in the sample, we calculated odds ratios (ORs) and then used a correction method [32] to determine prevalence ratios (PRs) [33]. PRs were converted from the ORs from the logistic regression models and their confidence intervals using the following function: PR = OR/(1 − P0 + (P0 × OR)) where OR is the odds ratio and P0 is the proportion of incidence in the outcome variable for the reference group. We computed PR and 95% confidence intervals (95%CI) of CMR across quartiles of diet exposures, using the first quartile as the reference. All models were adjusted for age and sex, as well as quartiles of the most representative variable of the other clusters (model 1), and then additionally adjusted for geographical region of employment, education, work hours, and sleep duration (model 2).

We computed p-values for linear trends across exposure quartiles using each category median values, respectively. All statistical tests had a significance threshold of 0.05. We also performed stratified analyses by sex, age (median split), and physical activity (low to moderate vs. high) and evaluated the statistical significance of potential heterogeneity across strata by addition of the cross-term product to the main effect model and reporting the p-value based on a log likelihood ratio test. In this AHMS cohort, there is a strong and significant bias towards under-reporting in individuals with a higher BMI [18]. Therefore, we also conducted a subgroup analysis, by restricting to only individuals with a BMI ≥ 25 kg/m^2^ and further adjusting for BMI, in order to determine if there was residual confounding. Finally, in sensitivity analysis, we also restricted our outcome definition to impaired blood glucose control only, as defined above, since insulin resistance may underlie the other factors of CMR [34].

## 3. Results

### 3.1. Sample Characteristics

Sample characteristics, overall and stratified by sex, are presented in Table 1. Briefly, the mean age of participants was 40.8 (standard deviation [SD] = 9.1), more male (61.7%) and predominantly white (97.3%). On average, the population was overweight with a mean BMI of 27.0 kg/m^2^ (SD = 4.1). More males reported working greater than 40 hours per week (63.7%) than females (37.0%).

### 3.2. Diet Exposures Selected by K-means Cluster Analysis

**Figure 1:**
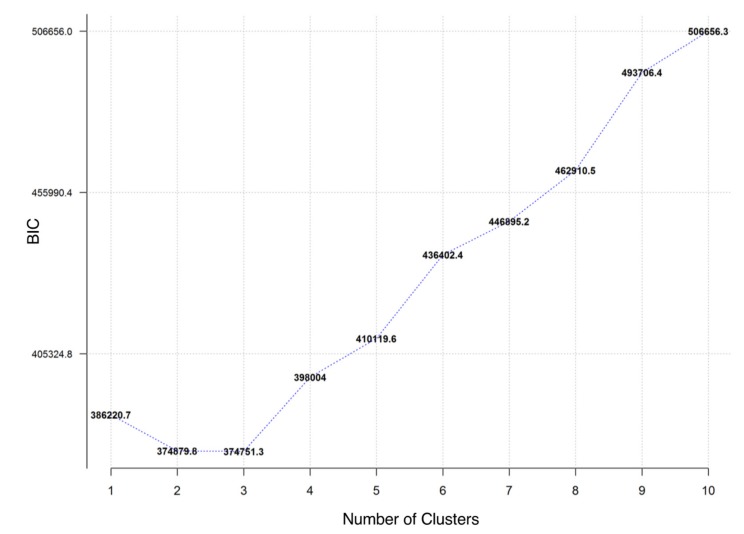
K-means optimal cluster determination. The lowest Bayesian Information Criterion (BIC) indicates the best fit model and thus the optimal number of clusters for k-means clustering analysis.

Based on the lowest BIC with the minimal number of clusters (Figure 1), we retained three clusters as optimum for the primary k-means clustering analysis. Cluster 1 primarily consisted of measures of energy-adjusted fat intake variables and energy intake variables (see Table S2 for variable cluster assignments and silhouette values). Average energy-adjusted saturated fat intake had the highest silhouette value (0.27) and was therefore the most representative diet exposure for Cluster 1. Cluster 2 primarily consisted of energy-adjusted carbohydrate intake variables and meal frequency variables. Average energy-adjusted carbohydrate intake had the highest silhouette value (0.22) and was therefore selected as the most representative diet exposure for Cluster 2. Cluster 3 primarily consisted of average energy-adjusted protein intake variables and irregularity scores. Average energy-adjusted protein intake had the highest silhouette value (0.14) and was therefore selected as the most representative diet exposure for Cluster 3. Descriptive statistics for the top hit variables of Clusters 1, 2, and 3 are given in Table 2.

**Figure 2:**
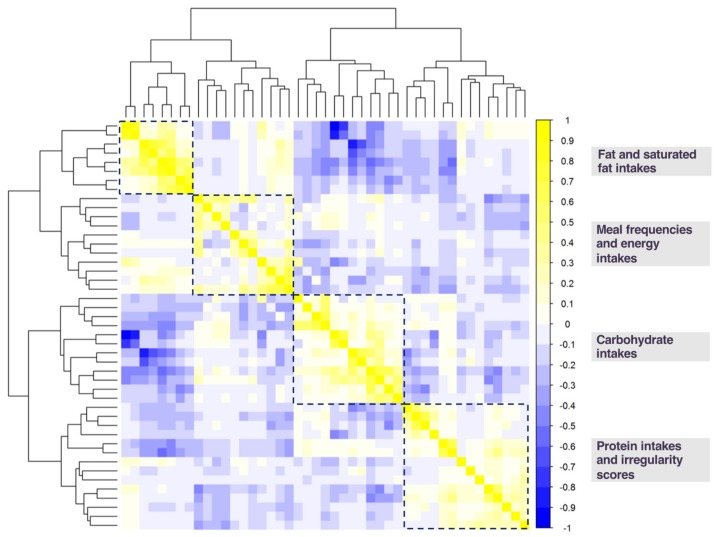
Dendrogram and correlation matrix of diet exposures. Variables are ordered by best fit to clusters determined by hierarchical clustering. The four-cluster solution is outlined in dashed lines. Yellow indicates a positive correlation coefficient; blue indicates a negative correlation coefficient. Variable order from top to bottom and left to right: (1) average fat for breakfast; (2) average saturated fat for breakfast; (3) average fat for lunch; (4) average saturated fat for lunch; (5) average daily fat intake; (6) average daily saturated fat intake; (7) average fat for dinner; (8) average saturated fat for dinner; (9) average daily eating occasions; (10) dinner frequency; (11) breakfast frequency; (12) lunch frequency; (13) late night and early morning snack frequency; (14) average calories for snacks; (15) evening snack frequency; (16) average calories for breakfast; (17) average calories for dinner; (18) average calories for lunch; (19) average daily caloric intake; (20) average fiber for breakfast; (21) average fiber for dinner; (22) average fiber for lunch; (23) average daily fiber intake; (24) average carbohydrates for breakfast; (25) average sugar for breakfast; (26) average carbohydrates for lunch; (27) average sugar for lunch; (28) average daily carbohydrate intake; (29) average daily sugar intake; (30) average carbohydrates for dinner; (31) average sugar for dinner; (32) average protein for lunch; (33) average daily protein intake; (34) average protein for dinner; (35) average protein for breakfast; (36) saturated fat irregularity; (37) fat irregularity; (38) breakfast irregularity; (39) lunch irregularity; (40) dinner irregularity; (41) sugar irregularity; (42) carbohydrate irregularity; (43) protein irregularity; (44) fiber irregularity; (45) daily caloric irregularity.

Figure 2 shows the resultant dendrogram and correlation matrix of the variables ordered by hierarchical clustering. The dendrogram illustrates well two clusters that split at a comparable distance from the first node, creating four clusters and the dendrogram does not clearly indicate a three-cluster solution for this variable set. This suggests that four clusters may be the next best model fit for the correlation structure across our variable exposure set. A four-cluster solution is further supported by the correlation matrix between variables (Figure 2). As shown in Figure 2, cross-correlation across all variables and between the three-cluster solution shows that within each cluster, variables are not necessarily cohesive, as compared to the correlation matrix observed for the four-cluster solution. This impression is further endorsed by the use of alternative indices of the optimal cluster number generated by the NbClust package. Of the 21 indices used, 12 indicated an optimal number of clusters between 2 and 4 clusters (see Figure S1 for a distribution of the NbClust index results). Together, this converging line of results support a four-cluster solution to k-means clustering (see Table S3 for variable cluster assignment and silhouette values), and we use the results of this four-cluster solution to guide further regression analyses.

**Figure 3:**
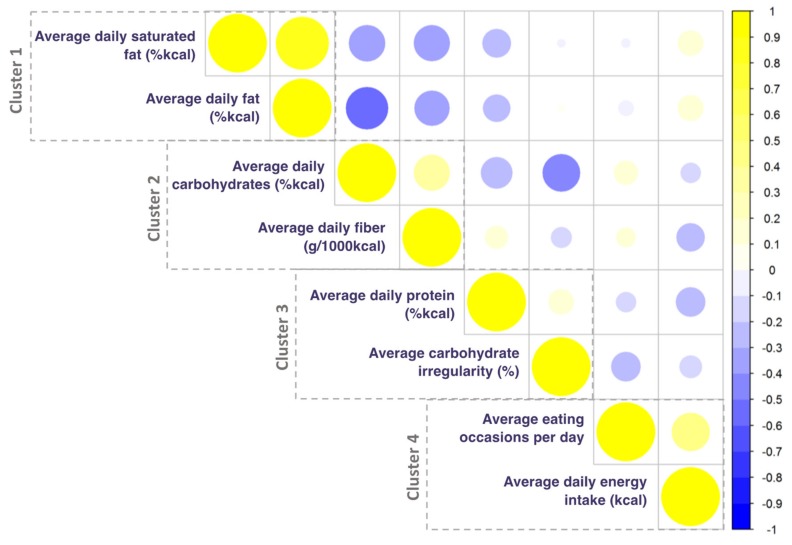
Correlation matrix of top two diet exposures for k-means 4- cluster solution. Yellow indicates a positive correlation coefficient; blue indicates a negative correlation coefficient.

In order to maximize explanatory power of our next analytical step and identify potential collinearity of respective top hit variables, we probed the correlation between the two top diet exposures of each of the four clusters (Figure 3). Results showed low correlations between the first and second hit variables of clusters 2, 3, and 4 (<0.50, so that we retained these second hit variables for subsequent analysis as well). In summary, the four-cluster solution consisted of an (i) energy-adjusted fat intake cluster; (ii) energy-adjusted carbohydrate intake cluster; (iii) energy-adjusted protein intake and irregularity score cluster; and (iv) meal frequency and energy intake cluster. The variables with the highest silhouette values in each four clusters were (i) average energy-adjusted saturated fat intake; (ii) average energy-adjusted carbohydrate intake; (iii) average energy-adjusted protein intake; and (iv) average eating occasions per day (with the silhouette values of 0.32, 0.23, 0.15, and 0.15, respectively). The second hit variables for clusters 2, 3, and 4 were (i) average fiber intake; (ii) average carbohydrate irregularity; and (iii) average energy intake (with silhouette values of 0.18, 0.12, and 0.14, respectively).

### 3.3. Diet Exposure Association with Cardiometabolic Risk

There were 2912 prevalent cases of increased CMR in the AHMS cohort. In the three-cluster model solution, we observed that individuals with highest energy-adjusted carbohydrate intake had a 21% lower likelihood of CMR than those with the lowest carbohydrate intake levels (Table 2; PR = 0.79, 95% CI: 0.71 to 0.86; *p_trend_* < 0.001). Further covariate adjustment did not attenuate responses. However, we found there to be no cross-sectional associations between energy-adjusted saturated fat intake or energy-adjusted protein intake and CMR (Table 2).

We observed in further regressions analyses including the four-cluster k-means solution that individuals with the highest levels energy-adjusted carbohydrate intake (on average 56% of energy intake) had a lower likelihood of CMR than those participants with the lowest carbohydrate intake (PR = 0.89, 95% CI = 0.81–0.98; *p_trend_* = 0.02). Individuals with the highest fiber intake (on average 13.30 g per 1000 kcal) had a 24% lower likelihood of CMR than those with the lowest fiber intakes (PR = 0.76, 95% CI = 0.68–0.85; *p_trend_* < 0.001) We also observed that individuals who ate most frequently (i.e., on average 5 meals a day with >50 kcal) throughout the day had a lower likelihood of CMR than those eating less frequent meals (3 meals/day; PR = 0.76, 95% CI: 0.68–0.85; *p_trend_* < 0.001). Further covariate adjustment did not attenuate responses (Table 3). We did not observe any other significant cross-sectional associations between the data-driven identified diet exposures (i.e., average energy intake, energy adjusted intakes of protein and fat and average carbohydrate irregularity) and likelihood of CMR.

### 3.4. Post Hoc Analyses

Results of stratified regression analyses by sex, age (median split), and physical activity (low to moderate vs. high) can be found in  Tables S4–6. We found there to be a protective effect of higher carbohydrate intake on CMR in men but not women (*p_interaction_* < 0.001; Table S4). Men with the highest carbohydrate intakes were at a 20% lower likelihood of CMR (PR = 0.80, 95%CI = 0.70–0.89; Table S4). Similarly, we found a protective effect of higher fiber intake on CMR in men but not women (*p_interaction_* = 0.02; Table S4). Our findings also indicate a sex-specific association of carbohydrate intake irregularity on CMR (*p_interaction_* = 0.01; Table S4); however, there is no significant main effect. Additionally, we found there to be an age-specific association between carbohydrate intake and CMR, although again the main effects were not significant (Table S5). Associations between CMR and diet exposures were not dependent on physical activity level (Table S6). In subgroup analysis of individuals with BMI ≥ 25 kg/m^2^, higher fiber intake and meal frequency were still associated with lower likelihood of CMR (*p_trend_* = 0.009 and 0.01, respectively; Table S7). We also found there to be a protective effect of highest protein intake (on average 21% of daily energy) on CMR in individuals with overweight status (PR = 0.88, 95%CI = 0.78–0.99; Table S7).

In sensitivity analyses, we examined the association between diet exposures and impaired blood glucose control. Results of this analysis can be found in Table S8. About 42% of CMR cases had impaired blood glucose control (i.e., HbA1c levels ≥ 5.7%). Analyses restricting our outcome to impaired blood glucose control showed that individuals with highest saturated fat intake (on average 15% of energy intake) had a 19% higher likelihood of impaired blood glucose control than participants with the lowest saturated fat intake (PR = 1.19; 95%CI = 1.08–1.29; *p_trend_* < 0.001). We also observed that individuals with highest carbohydrate intakes had an 8% higher likelihood of impaired blood glucose control compared to those individuals with lowest carbohydrate intakes (PR = 1.08; 95%CI = 0.99–1.17; *p_trend_* = 0.02). We did not observe any other significant cross-sectional associations between the data-driven identified diet exposures (i.e., average energy intake, eating occasions per day, energy adjusted intake of protein and carbohydrate irregularity) and likelihood of impaired blood glucose control.

## 4. Discussion

In this study we systematically analyzed the association between non-redundant diet exposures and CMR in the AHMS, a large, on-going cohort study. We implemented dimension reduction techniques to select diet exposure variables among a large set of a priori defined candidate exposures, expected to be relevant for cardiometabolic health. Dimension reduction techniques enabled a data-driven selection of exposures among often highly correlated variables; we then used the diet exposures that were identified as the most representative of overall diet intake patterns. Out of 45 candidate variables derived from 7-day food diaries, seven diet exposures were ultimately identified as non-redundant diet exposures, of which energy-adjusted carbohydrate intake, fiber intake, and meal frequency were associated with CMR.

We found higher energy-adjusted intake of carbohydrates to be associated with lower CMR. Findings from previous studies also examining this association are somewhat mixed [35]. For example, in a two-year study of 322 obese individuals assigned to a low fat, low carbohydrate or Mediterranean diet, the individuals on a low carbohydrate diet lost more weight and had improved lipids when compared to the those on a low-fat diet [36]. However, the same study also observed benefits in individuals assigned to a Mediterranean diet, typically higher in carbohydrate intake, with individuals seeing increased weight loss and improved insulin and fasting glucose levels on this diet. These findings would rather suggest that diets, such as the Mediterranean Diet, which include higher intake of high quality carbohydrates, such as whole grains and increased fiber intake, may be beneficial for cardiometabolic disease risk factors [37,38,39]. We also found higher carbohydrate intake to be associated with higher risk of impaired glucose control, arguably the most important risk factor for CMR. These findings are consistent with a recent meta-analysis of 10 different trials that found a 34% reduction in HbA1c in individuals after 1 year on a low carbohydrate diet [40]. However, the link between higher carbohydrate intake and higher glucose levels is more established for carbohydrates with a higher glycemic load (i.e., low-quality carbohydrates) [41]. We also observed that individuals with the highest fiber intake (on average 13.30 g/1000 kcal) had a lower likelihood of CMR, and upon adjustment for fiber intake the protective effect of carbohydrate intake on CMR was somewhat attenuated. This suggests that fiber intake may be a potential mediating factor in the association between carbohydrate intake and CMR. Therefore, further analysis of the quality of carbohydrates captured by the carbohydrate dimension we analyzed would be of future interest.

Another possible explanation for our findings is that those individuals with the lowest carbohydrate intake (consisting, on average, of 39% of daily energy intake) may be intentionally trying to reduce carbohydrate intake because they are aware of their CMR status. Indeed, the average carbohydrate intake in the lowest quartile falls below CDC recommendations for individuals with diabetes (45%) [42] and within the range (10–40%) some studies have shown to be beneficial for metabolic syndrome [43,44,45]. Additionally, Seidelmann et al. [46] have suggested a U-shaped relationship for carbohydrate intake with minimal risk for all-cause mortality observed when carbohydrate intake constituted 50–55% of energy intake. In our study, individuals with the highest carbohydrate intakes—consisting on average of 56%—fall just above this range. In fact this value falls within the range for recommended carbohydrate intake (45–65%), so these highest carbohydrate eaters may be those that are following dietary guidelines [47]. Our findings do not allow us to disentangle effects of disease status on diet from those of diet on metabolic disease status, given our cross-sectional study design, warranting prospective studies to address this question further.

Associations between meal frequency and CMR are similarly complex. Many previous studies have found that increased meal frequency is associated with increased energy intake [48,49,50,51]. We observe in our study that those individuals that eat most frequently (>5 meals/day) also have the greatest energy intake. However, these individuals that ate most frequently also had the lowest energy intake, on average, per eating occasion. Eating more frequent, less caloric meals therefore may be beneficial to CMR. This has also been shown in a study conducted within the Norfolk cohort of EPIC (*N* = 14,666), where greater meal frequency (>6 meals/day) improved LDL and cholesterol levels [52]. Another study of 2696 individuals of the INTERnational study on Macro/micronutrients and blood pressure (INTERMAP) found increased eating frequency (>6 meals/day) to be associated with improved diet quality and lower BMI [53]. Despite this, the alternative explanation of reverse causation could again be true in this case: those individuals that are eating fewer meals per day are aware of their risk status and therefore consciously trying to eat less overall.

Our data-driven approach should be considered hypothesis-generating and provides novel paths towards addressing the multi-dimensionality in exposure information. It is noteworthy that dimension reduction method results are dependent on the underlying data structure and may be of limited generalizability. Data formats, study sample size, and interpretability will have to be considered when choosing specific dimension reduction methods, including k-means clustering, for future research studies. Our findings highlight the benefits of such dimension reduction approaches when handling high-dimensional data, including nutritional exposure data. The application to nutrient intake patterns is novel and future studies embedded in distinct cohort studies and populations will be useful to delineate generalizability and uncover differences in dietary intake patterns. In addition, future studies are needed to understand the usefulness of our approach in guiding prevention and intervention strategies. Our data clearly highlight the potential of systematically considering all potentially relevant exposures, as well as the need to consider diet exposure dimensions like frequency, temporality, and irregularity in dietary intake. Further, these data-driven approaches may pave the way for future studies that would systematically expand dietary factors to all nutrients, including vitamins, minerals, and other dimensions of dietary intake, including diet quality. Our data-driven approach allows to include multiple dietary exposures in analyses concurrently and may help to understand independent contributions to disease risk among a host of candidate variables. This in turn may help inform dietary intervention strategies.

Our study has several limitations. First, participants self-reported dietary intake. Bias in reporting is common for all dietary recording methods and in this AHMS cohort, there is a strong and significant bias towards under-reporting in individuals with a higher BMI [18]. However, our primary results were not attenuated upon restriction to those individuals with higher BMI. Further, dietary recording was limited to 7 days and may not reflect habitual intake. The balance of data collection and increased participant burden needs to be considered in collection periods greater than 7 days. Worth noting, however, is that the 7-day diary captures weekday/weekend variation in intake and includes information on other aspects of diet. An additional limitation of this present study includes limited reports for shift work timing. Shift work can greatly influence diurnal eating patterns, particularly the temporal distribution of certain macronutrient intake [54]. However, preliminary findings indicate that in the AHMS cohort, duration of weekly working hours (which we have adjusted for in this present study) has been found to be associated with shift work, where those individuals with the highest weekly working duration are the most likely to participate in shift work. Finally, we did not have precise timing information for eating occasions. However, we were able to approximate temporal distribution of intake by using the pre-specified categories for eating occasions included in the food diaries. We also derived one variable, frequency of having both a late night and early morning snack, in order to estimate fasting period, although this did not emerge as a relevant dimension in our analysis. This allows for the possibility for future analyses of diurnal eating patterns and cardiometabolic health, as the temporal aspect of eating patterns is becoming an established risk factor for metabolic diseases [27].

The present study also has several strengths. The use of 7-day food diaries enabled for a detailed weekly dietary intake to be measured. Using this high-quality dietary intake data, we were able to rigorously and systematically analyze multiple aspects of diet exposures using dimension reduction techniques to prioritize non-redundant diet exposures that may most strongly influence CMR. Our approach demonstrates the power of data-driven approaches for diet exposure selections when a host of correlated diet exposures are hypothesized to influence cardiometabolic health. Similar techniques have been used in previous studies, where dimension reduction techniques are used to cluster individuals based upon their dietary habits [25,26,27,55] to see how they may be differentially affected by chronic disease risk. Our approach takes a multi-dimensional approach by allowing us to quantify several dimensions of dietary intake that include temporality, regularity, and macronutrient composition. Understanding which of these dimensions may represent independent clusters of dietary intakes may then allow for multi-target interventions of specific dietary aspects.

## Figures and Tables

**Table 1:** Baseline characteristics stratified by sex in the Airwave Health Monitoring Study cohort (*N* = 8090). Values are presented as mean (standard deviation) or absolute counts and percentages.

		Overall	Male	Female
Age, years		40.8 (9.1)	41.9 (8.8)	39.0 (9.4)
Body mass index, kg/m^2^		27.0 (4.1)	27.7 (3.6)	25.7 (4.6)
Ethnicity	White	7874 (97.3%)	96.9%	98.0%
	Other	203 (2.5%)	2.9%	1.9%
	Missing	13 (0.2%)	0.2%	0.2%
Region of employment	England	5822 (72.0%	70.5%	74.3%
	Scotland	1333 (16.5%)	18.4%	13.3%
	Wales	792 (9.8%)	9.5%	10.3%
	Missing	143 (1.8%)	1.6%	2.1%
Educational level	A levels/Higher or equivalent	2599 (32.1%)	32.0%	32.4%
	Bachelor’s degree or higher	2222 (27.5%)	27.5%	30.2%
	Other	3268 (40.4%)	42.3%	37.3%
	Missing	1 (0%)	0%	0%
Annual household income	Less than £37,999	2123 (26.2%)	19.8%	36.6%
	£38,000 to £77,999	5162 (63.8%)	70.4%	53.1%
	More than £78,000	804 (9.9%)	9.7%	10.3%
	Missing	1 (0%)	0%	0%
Relationship status	Married/cohabitating	6411 (79.2%)	85.8%	68.8%
	Single	876 (10.8%)	6.5%	17.9%
	Divorced/separated	619 (7.7%)	6.4%	9.7%
	Other	183 (2.3%)	1.4%	3.6%
	Missing	1 (0%)	0%	0%
Work hours	Less than/equal to 40 hours/week	3766 (46.6%)	36.3%	63.0%
	Greater than 40 hours/week	4324 (53.4%)	63.7%	37.0%
	Missing	1 (0%)	0%	0%
Physical activity *	Low	1561 (19.3%)	17.3%	22.4%
	Moderate	5593 (69.1%)	70.4%	67.0%
	High	936 (11.6%)	12.2%	10.5%
	Missing	1 (0%)	0%	0%
Smoker status	Current	634 (7.8%)	6.6%	9.8%
	Former	1874 (23.2%)	23.3%	22.9%
	Never	5582 (69.0%)	70.1%	67.3%
	Missing	1 (0%)	0%	0%
Alcohol consumption	Yes	7479 (92.4%)	93.8%	90.2%
	No	611 (7.6%)	6.2%	9.8%
	Missing	0 (0%)	0%	0%
Sleep duration	Less than 7 hours	2563 (31.7%)	34.4%	27.3%
	7–8 hours	5262 (65.0%)	63.1%	68.2%
	9 hours or more	264 (3.3%)	2.5%	4.5%
	Missing	1 (0%)	0%	0%

^*^ Scored using the IPAQ Scoring Guidelines for vigorous, moderate and low activity categories.

**Table 2:** Cross-sectional associations between k-means three-cluster solution top hit diet exposure quartiles with cardiometabolic risk prevalence (*N* = 8090). Values are presented as prevalence ratio (95% confidence interval). Significant p-values (*p* <0.05) are marked in bold.

Cluster 1: Saturated Fat Intake (%kcal ^*^)					
	Quartile 1	Quartile 2	Quartile 3	Quartile 4	*P_trend_*
Mean (SD ^†^)	8 (1)	11 (0)	12 (0)	15 (2)	
Interquartile Range	3–9	10–11	12–13	14–30	
Prevalent Cases/N	478/1443	702/2054	822/2233	910/2360	
Model 1	1.00 (ref)	1.01 (0.91; 1.11)	1.06 (0.96; 1.17)	1.06 (0.95; 1.17)	0.27
Model 2	1.00 (ref)	1.01 (0.91; 1.11)	1.05 (0.95; 1.16)	1.05 (0.94; 1.16)	0.37
**Cluster 2: Carbohydrate Intake (%kcal)**					
	**Quartile 1**	**Quartile 2**	**Quartile 3**	**Quartile 4**	***P_trend_***
Mean (SD)	39 (4)	46 (1)	49 (1)	56 (4)	
Interquartile Range	10–43	44–47	48–51	52–76	
Prevalent Cases/N	851/1955	668/1804	665/1918	728/2413	
Model 1	1.00 (ref)	0.88 (0.80; 0.95)	0.85 (0.77; 0.92)	0.79 (0.71; 0.86)	**<0.001**
Model 2	1.00 (ref)	0.88 (0.81; 0.96)	0.85 (0.78; 0.93)	0.79 (0.71; 0.86)	**<0.001**
**Cluster 3: Protein Intake (%kcal)**					
	**Quartile 1**	**Quartile 2**	**Quartile 3**	**Quartile 4**	***P_trend_***
Mean (SD)	13 (1)	16 (0)	17 (0)	21 (3)	
Interquartile Range	8–14	15–16	17–18	19–55	
Prevalent Cases/N	437/1344	771/2144	820/2162	884/2440	
Model 1	1.00 (ref)	1.05 (0.95; 1.16)	1.07 (0.97; 1.18)	1.02 (0.91; 1.13)	0.83
Model 2	1.00 (ref)	1.05 (0.95; 1.16)	1.07 (0.97; 1.18)	1.02 (0.91; 1.13)	0.82

Model 1: adjusted for age and sex, as well as for the quartiles of the most representative variable of the other clusters, respectively. Model 2: adjusted for covariates of Model 1, plus education level, region of employment, work hours and sleep duration. ^*^ kilocalorie, ^†^ standard deviation.

**Table 3:** Cross-sectional associations between k-means four-cluster solution top hit diet exposure quartiles with cardiometabolic risk prevalence (*N* = 8090). Values are presented as prevalence ratio (95% confidence interval). Significant p-values (*p* < 0.05) are marked in bold.

Cluster 1: Saturated Fat Intake (%kcal ^*^)					
	Quartile 1	Quartile 2	Quartile 3	Quartile 4	*P_trend_*
Mean (SD ^†^)	8 (1)	11 (0)	12 (0)	15 (2)	
Interquartile Range	3–9	10–11	12–13	14–30	
Prevalent Cases/N	478/1443	702/2054	822/2233	910/2360	
Model 1	1.00 (ref)	0.99 (0.89; 1.10)	1.02 (0.92; 1.13)	1.01 (0.90; 1.12)	0.87
Model 2	1.00 (ref)	0.99 (0.89; 1.10)	1.02 (0.92; 1.13)	1.00 (0.89; 1.12)	0.95
**Cluster 2: Carbohydrate Intake (%kcal)**					
	**Quartile 1**	**Quartile 2**	**Quartile 3**	**Quartile 4**	***P_trend_***
Mean (SD)	39 (4)	46 (1)	49 (1)	56 (4)	
Interquartile Range	10–43	44–47	48–51	52–76	
Prevalent Cases/N	851/1955	668/1804	665/1918	728/2413	
Model 1	1.00 (ref)	0.93 (0.85; 1.01)	0.93 (0.84; 1.01)	0.89 (0.81; 0.98)	**0.02**
Model 2	1.00 (ref)	0.93 (0.85; 1.01)	0.92 (0.84; 1.01)	0.89 (0.80; 0.98)	**0.01**
**Cluster 2: Fiber Intake (g/1000 kcal)**					
	**Quartile 1**	**Quartile 2**	**Quartile 3**	**Quartile 4**	***P_trend_***
Mean (SD)	6.32 (0.92)	8.33 (0.48)	10.09 (0.57)	13.30 (2.09)	
Interquartile Range	2.54—7.50	7.51—9.16	9.17—11.14	11.15—35.62	
Prevalent Cases/N	806/2020	757/2024	688/2019	661/2027	
Model 1	1.00 (ref)	0.91 (0.84; 0.99)	0.81 (0.73; 0.89)	0.76 (0.68; 0.85)	**<0.001**
Model 2	1.00 (ref)	0.92 (0.84; 1.00)	0.82 (0.74; 0.90)	0.78 (0.70; 0.86)	**<0.001**
**Cluster 3: Eating occasions/day**					
	**Quartile 1**	**Quartile 2**	**Quartile 3**	**Quartile 4**	***P_trend_***
Mean (SD)	3.06 (0.35)	3.8 (0.16)	4.35 (0.16)	5.17 (0.44)	
Interquartile Range	1.71—3.43	3.57–4.00	4.14—4.57	4.71—7.71	
Prevalent Cases/N	649/1747	819/2150	755/2013	689/2162	
Model 1	1.00 (ref)	0.99 (0.90; 1.08)	0.94 (0.85; 1.03)	0.76 (0.68; 0.85)	**<0.001**
Model 2	1.00 (ref)	0.99 (0.90; 1.08)	0.94 (0.85; 1.04)	0.76 (0.68; 0.86)	**<0.001**
**Cluster 3: Energy Intake (kcal)**					
	**Quartile 1**	**Quartile 2**	**Quartile 3**	**Quartile 4**	***P_trend_***
Mean (SD)	1374.52 (174.91)	1759.66 (85.25)	2056.47 (91.38)	2596.07 (330.91)	
Interquartile Range	627.33—1603.69	1603.91—1906.23	1906.24—2227.87	2227.97—4620.40	
Prevalent Cases/N	678/2023	716/2022	763/2022	755/2023	
Model 1	1.00 (ref)	0.96 (0.87; 1.06)	0.96 (0.86; 1.06)	0.95 (0.84; 1.07)	0.49
Model 2	1.00 (ref)	0.97 (0.88; 1.07)	0.97 (0.87; 1.07)	0.96 (0.85; 1.08)	0.64
**Cluster 4: Protein Intake (%kcal)**					
	**Quartile 1**	**Quartile 2**	**Quartile 3**	**Quartile 4**	***P_trend_***
Mean (SD)	13 (1)	16 (0)	17 (0)	21 (3)	
Interquartile Range	8–14	15–16	17–18	19–55	
Prevalent Cases/N	437/1344	771/2144	820/2162	884/2440	
Model 1	1.00 (ref)	1.06 (0.96; 1.17)	1.08 (0.97; 1.19)	1.02 (0.91; 1.14)	0.86
Model 2	1.00 (ref)	1.06 (0.96; 1.17)	1.08 (0.97; 1.19)	1.02 (0.91; 1.14)	0.88
**Cluster 4: Carbohydrate Irregularity (%)**					
	**Quartile 1**	**Quartile 2**	**Quartile 3**	**Quartile 4**	***P_trend_***
Mean (SD)	7 (2)	11 (1)	15 (1)	23 (5)	
Interquartile Range	0–9	10 -12	13–17	18–79	
Prevalent Cases/N	573/1706	621/1782	919/2520	799/2082	
Model 1	1.00 (ref)	1.01 (0.92; 1.12)	1.05 (0.95; 1.14)	1.04 (0.94; 1.14)	0.39
Model 2	1.00 (ref)	1.01 (0.91; 1.11)	1.05 (0.95; 1.14)	1.03 (0.93; 1.14)	0.43

Model 1: adjusted for age and sex, as well as for the quartiles of the most representative variable of the other clusters, respectively. Model 2: adjusted for covariates of Model 1, plus education level, region of employment, work hours and sleep duration. ^*^kilocalorie, ^†^standard deviation.

## Supplementary Materials

The following are available online at https://www.mdpi.com/2072-6643/12/4/1170/s1, Figure S1: Frequency of recommended number of clusters determined by 21 indices used in the R package NbClust. Used to determine optimal number of clusters for k-means clustering, Table S1: List of diet variables derived to be used for k-means clustering analyses, Table S2: Cluster assignments and silhouette coefficient for three-cluster solution to k-means clustering. Top hit variables are bolded, Table S3: Cluster assignments and silhouette coefficient for four-cluster solution to k-means clustering. Top hit variables are bolded, Table S4: Cross-sectional associations between k-means four-cluster solution top hit diet exposure quartiles with cardiometabolic risk prevalence stratified by sex (*Nmale* = 4992, *Nfemale* = 3098). Values are presented as prevalence ratio (95% confidence interval). Significant *p*-values (*p* < 0.05) are marked in bold, Table S5: Cross-sectional associations between k-means four-cluster solution top hit diet exposure quartiles with cardiometabolic risk prevalence stratified by age (median split, *N* ≤ 41 years = 4133, *N* > 41 years = 3957). Values are presented as prevalence ratio (95% confidence interval). Significant p-values (*p* < 0.05) are marked in bold, Table S6: Cross-sectional associations between k-means four-cluster solution top hit diet exposure quartiles with cardiometabolic risk prevalence stratified by physical activity (*Nlow activity* = 7154, *Nhigh activity* = 936). Values are presented as prevalence ratio (95% confidence interval). Significant p-values (*p* < 0.05) are marked in bold, Table S7 Cross-sectional associations between k-means four-cluster solution top hit diet exposure quartiles with cardiometabolic risk prevalence within overweight individuals (body mass index [BMI] ≥ 25 kg/m2, *N* = 5416). Values are presented as prevalence ratio (95% confidence interval). Significant p-values (*p* < 0.05) are marked in bold, Table S8: Cross-sectional associations between k-means four-cluster solution top hit diet exposure quartiles with impaired blood glucose control prevalence (*N* = 8090). Values are presented as prevalence ratio (95% confidence interval). Significant p-values (*p* < 0.05) are marked in bold.
