# Supplementary material for: Quantifying Diet Intake and Its Association with Cardiometabolic Risk in the UK Airwave Health Monitoring Study: A Data-Driven Approach
Source: Nutrients. 2020 Apr 22;12(4):1170. doi: 10.3390/nu12041170 (PMC7230946; doi:10.3390/nu12041170)
Supplement: Supplementary file 1 [file nutrients-12-01170-s001.pdf]

Supplementary Materials Hunt et al.

Table S1. List of diet variables derived to be used for k-means clustering analyses.

**Variable**	**Units**
Average daily caloric intake	kcal/day
Average daily protein intake	%kcal/day
Average daily carbohydrate intake	%kcal/day
Average daily sugar intake	%kcal/day
Average daily fiber intake	g/1000kcal
Average daily fat intake	%kcal/day
Average daily saturated fat intake	%kcal/day
Average eating occasions/day	#
Breakfast frequency	% (Yes)
Lunch frequency	% (Yes)
Dinner frequency	% (Yes)
Late night snack frequency	% (Yes)
Late night *and* early morning snack frequency	% (Yes)
Average calories for breakfast	kcal
Average calories for lunch	kcal
Average calories for dinner	kcal
Average calories for snacks	kcal
Average protein for breakfast	%kcal/meal
Average protein for lunch	%kcal/meal
Average protein for dinner	%kcal/meal
Average carbohydrate for breakfast	%kcal/meal
Average carbohydrate for lunch	%kcal/meal
Average carbohydrate for dinner	%kcal/meal
Average sugar for breakfast	%kcal/meal
Average sugar for lunch	%kcal/meal
Average sugar for dinner	%kcal/meal
Average fiber for breakfast	g/1000kcal
Average fiber for lunch	g/1000kcal
Average fiber for dinner	g/1000kcal
Average fat for breakfast	%kcal/meal
Average fat for lunch	%kcal/meal
Average fat for dinner	%kcal/meal
Average saturated fat for breakfast	%kcal/meal
Average saturated fat for lunch	%kcal/meal
Average saturated fat for dinner	%kcal/meal
Breakfast irregularity score	%
Lunch irregularity score	%
Dinner irregularity score	%
Daily caloric irregularity score	%
Protein irregularity score	%
Carbohydrate irregularity score	%
Sugar irregularity score	%
Fiber irregularity score	%
Fat irregularity score	%
Saturated fat irregularity score	%

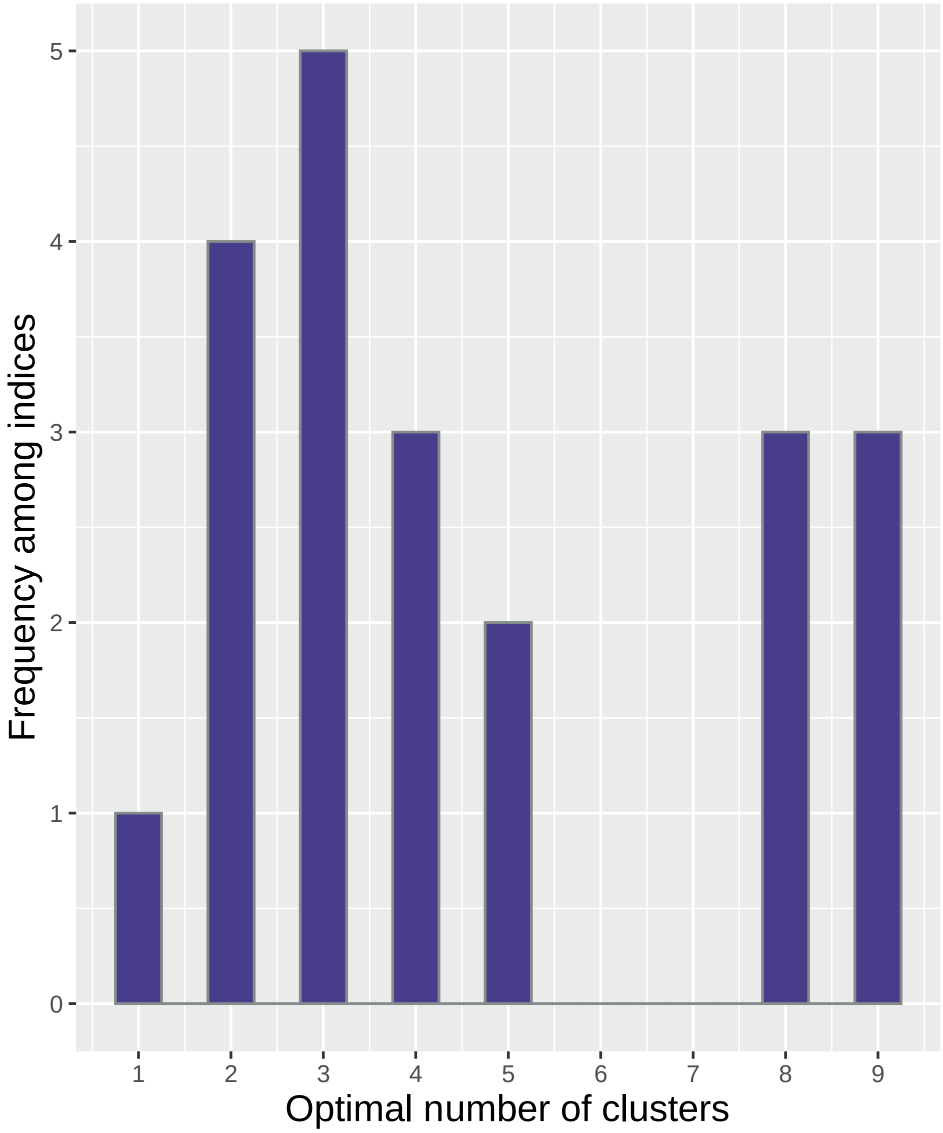

Figure S1. Frequency of recommended number of clusters determined by 21 indices used in the R package NbClust. Used to determine optimal number of clusters for k-means clustering.

Table S2. Cluster assignments and silhouette coefficient for three-cluster solution to k-means clustering. Top hit variables are bolded.

Variable	Cluster	Silhouette
**Average daily saturated fat intake**	**1**	**0.27**
**Average daily fat intake**	**1**	**0.26**
Average fat for lunch	1	0.21
Average saturated fat for lunch	1	0.21
Average saturated fat for dinner	1	0.18
Average fat for dinner	1	0.17
Average daily caloric intake	1	0.16
Average calories for lunch	1	0.15
Average calories for dinner	1	0.14
Average saturated fat for breakfast	1	0.13
Average calories for breakfast	1	0.12
Average fat for breakfast	1	0.12
Average calories for snacks	1	0.06
**Average daily carbohydrate intake**	**2**	**0.22**
**Average daily sugar intake**	**2**	**0.19**
Average carbohydrate for breakfast	2	0.14
Average carbohydrate for dinner	2	0.14
Average daily fiber intake	2	0.13
Average carbohydrate for lunch	2	0.12
Average sugar for dinner	2	0.11
Average sugar for breakfast	2	0.09
Average sugar for lunch	2	0.09
Breakfast frequency	2	0.08
Lunch frequency	2	0.07
Average fiber for lunch	2	0.06
Average eating occasions/day	2	0.06
Dinner frequency	2	0.04
Average fiber for dinner	2	0.04
Average fiber for breakfast	2	0.03
Late night snack frequency	2	0.00
Late night *and* early morning snack frequency	2	-0.01
**Average daily protein intake**	**3**	**0.14**
**Carbohydrate irregularity score**	**3**	**0.14**
Protein irregularity score	3	0.11
Daily caloric irregularity score	3	0.11
Fat irregularity score	3	0.11
Average protein for lunch	3	0.10
Sugar irregularity score	3	0.10
Fiber irregularity score	3	0.10
Saturated fat irregularity score	3	0.09
Average protein for dinner	3	0.09
Average protein for breakfast	3	0.07
Dinner irregularity score	3	0.06
Lunch irregularity score	3	0.05
Breakfast irregularity score	3	0.00

Table S3. Cluster assignments and silhouette coefficient for four-cluster solution to k-means clustering. Top hit variables are bolded.

Variable	Cluster	Silhouette
**Average daily saturated fat intake**	**1**	**0.32**
**Average daily fat intake**	**1**	**0.32**
Average saturated fat for lunch	1	0.21
Average saturated fat for dinner	1	0.21
Average fat for lunch	1	0.20
Average fat for dinner	1	0.20
Average saturated fat for breakfast	1	0.19
Average fat for breakfast	1	0.16
**Average daily carbohydrate intake**	**2**	**0.23**
**Average daily fiber intake**	**2**	**0.18**
Average carbohydrate for lunch	2	0.17
Average daily sugar intake	2	0.16
Average carbohydrate for dinner	2	0.14
Average carbohydrate for breakfast	2	0.14
Average sugar for lunch	2	0.13
Average sugar for dinner	2	0.11
Average fiber for lunch	2	0.11
Average sugar for breakfast	2	0.10
Average fiber for dinner	2	0.08
Average fiber for breakfast	2	0.05
**Average daily protein intake**	**3**	**0.15**
**Carbohydrate irregularity score**	**3**	**0.12**
Daily caloric irregularity score	3	0.10
Protein irregularity score	3	0.10
Average protein for dinner	3	0.10
Average protein for lunch	3	0.09
Fiber irregularity score	3	0.08
Sugar irregularity score	3	0.08
Fat irregularity score	3	0.08
Saturated fat irregularity score	3	0.07
Average protein for breakfast	3	0.06
Dinner irregularity score	3	0.06
Lunch irregularity score	3	0.05
Breakfast irregularity score	3	-0.02
**Average eating occasions/day**	**4**	**0.15**
**Average daily caloric intake**	**4**	**0.14**
Late night snack frequency	4	0.09
Average calories for snacks	4	0.07
Dinner frequency	4	0.04
Late night *and* early morning snack frequency	4	0.04
Lunch frequency	4	0.03
Breakfast frequency	4	0.01
Average calories for dinner	4	0.01
Average calories for breakfast	4	-0.01
Average calories for lunch	4	-0.01

Table S4. Cross-sectional associations between k-means four-cluster solution top hit diet exposure quartiles with cardiometabolic risk prevalence stratified by sex (N_{male} = 4992, N_{female} = 3098). Values are presented as prevalence ratio (95% confidence interval). Significant p-values (p < 0.05) are marked in bold.

		**Cluster 1: Saturated Fat Intake (%kcal*)**				
		**Quartile 1**	**Quartile 2**	**Quartile 3**	**Quartile 4**	$P_{interaction}$
Prevalent Cases / N	Male	328 / 864	506 / 1280	606 / 1426	622 / 1422	
	Female	150 / 579	196 / 774	216 / 807	288 / 938	
Model 1	Male	1.00 (ref)	0.99 (0.88; 1.11)	1.01 (0.90; 1.14)	0.96 (0.84; 1.09)	0.77
	Female	1.00 (ref)	1.00 (0.81; 1.21)	1.04 (0.85; 1.26)	1.13 (0.92; 1.37)	
Model 2	Male	1.00 (ref)	1.00 (0.88; 1.12)	1.01 (0.89; 1.13)	0.95 (0.83; 1.08)	0.78
	Female	1.00 (ref)	0.99 (0.81; 1.20)	1.04 (0.84; 1.26)	1.13 (0.91; 1.37)	
		**Cluster 2: Carbohydrate Intake (%kcal)**				
		**Quartile 1**	**Quartile 2**	**Quartile 3**	**Quartile 4**	$P_{interaction}$
Prevalent Cases / N	Male	649 / 1323	492 / 1155	481 / 1186	440 / 1328	
	Female	202 / 632	176 / 649	184 / 732	288 / 1085	
Model 1	Male	1.00 (ref)	0.92 (0.83; 1.01)	0.90 (0.81; 1.00)	0.80 (0.70; 0.89)	**<0.001**
	Female	1.00 (ref)	0.97 (0.80; 1.15)	0.99 (0.82; 1.19)	1.15 (0.95; 1.36)	
Model 2	Male	1.00 (ref)	0.92 (0.83; 1.01)	0.90 (0.81; 0.99)	0.78 (0.69; 0.88)	**<0.001**
	Female	1.00 (ref)	0.97 (0.81; 1.16)	0.99 (0.81; 1.18)	1.15 (0.95; 1.36)	
		**Cluster 2: Fiber Intake (g/1000kcal)**				
		**Quartile 1**	**Quartile 2**	**Quartile 3**	**Quartile 4**	$P_{interaction}$
Prevalent Cases / N	Male	629 / 1387	551 / 1294	486 / 1224	396 / 1087	
	Female	177 / 633	206 / 730	202 / 795	265 / 940	
Model 1	Male	1.00 (ref)	0.90 (0.82; 0.99)	0.82 (0.73; 0.91)	0.72 (0.63; 0.81)	**0.02**
	Female	1.00 (ref)	0.97 (0.80; 1.16)	0.82 (0.67; 0.99)	0.88 (0.71; 1.07)	
Model 2	Male	1.00 (ref)	0.91 (0.83; 1.00)	0.83 (0.74; 0.92)	0.74 (0.64; 0.84)	**0.02**
	Female	1.00 (ref)	0.97 (0.80; 1.16)	0.83 (0.68; 1.01)	0.89 (0.72; 1.08)	
		**Cluster 3: Eating Occasions / day**				
		**Quartile 1**	**Quartile 2**	**Quartile 3**	**Quartile 4**	$P_{interaction}$
Prevalent Cases / N	Male	478 / 1113	601 / 1361	523 / 1245	460 / 1273	
	Female	171 / 634	218 / 789	232 / 786	229 / 889	
Model 1	Male	1.00 (ref)	1.02 (0.92; 1.12)	0.95 (0.85; 1.06)	0.79 (0.69; 0.90)	0.38
	Female	1.00 (ref)	0.94 (0.77; 1.12)	0.91 (0.74; 1.10)	0.68 (0.54; 0.85)	
Model 2	Male	1.00 (ref)	1.02 (0.92; 1.12)	0.96 (0.85; 1.07)	0.80 (0.69; 0.91)	0.35
	Female	1.00 (ref)	0.92 (0.76; 1.11)	0.91 (0.74; 1.10)	0.68 (0.54; 0.84)	
		**Cluster 3: Energy Intake (kcal)**				
		**Quartile 1**	**Quartile 2**	**Quartile 3**	**Quartile 4**	$P_{interaction}$
Prevalent Cases / N	Male	320 / 692	460 / 1078	596 / 1445	686 / 1777	
	Female	358 / 1331	256 / 944	167 / 577	69 / 246	
Model 1	Male	1.00 (ref)	0.88 (0.78; 0.99)	0.85 (0.74; 0.96)	0.85 (0.74; 0.97)	0.06
	Female	1.00 (ref)	1.07 (0.92; 1.24)	1.19 (0.99; 1.40)	1.24 (0.97; 1.54)	
Model 2	Male	1.00 (ref)	0.89 (0.78; 1.00)	0.85 (0.74; 0.96)	0.86 (0.75; 0.98)	0.05
	Female	1.00 (ref)	1.09 (0.93; 1.26)	1.21 (1.01; 1.43)	1.25 (0.98; 1.56)	
		**Cluster 4: Protein Intake (%kcal)**				
		**Quartile 1**	**Quartile 2**	**Quartile 3**	**Quartile 4**	$P_{interaction}$
Prevalent Cases / N	Male	302 / 774	547 / 1324	595 / 1378	618 / 1516	
	Female	135 / 570	224 / 820	225 / 784	266 / 924	
Model 1	Male	1.00 (ref)	1.03 (0.92; 1.16)	1.03 (0.91; 1.16)	0.95 (0.83; 1.07)	0.96
	Female	1.00 (ref)	1.17 (0.96; 1.41)	1.21 (0.99; 1.46)	1.24 (1.01; 1.50)	
Model 2	Male	1.00 (ref)	1.04 (0.92; 1.16)	1.03 (0.91; 1.16)	0.95 (0.83; 1.07)	0.98
	Female	1.00 (ref)	1.17 (0.96; 1.41)	1.21 (0.99; 1.46)	1.25 (1.01; 1.51)	
		**Cluster 4: Carbohydrate Irregularity (%)**				
		**Quartile 1**	**Quartile 2**	**Quartile 3**	**Quartile 4**	$P_{interaction}$
Prevalent Cases / N	Male	392 / 1071	432 / 1064	647 / 1551	591 / 1306	
	Female	181 / 635	189 / 718	272 / 969	208 / 776	
Model 1	Male	1.00 (ref)	1.06 (0.94; 1.18)	1.08 (0.97; 1.19)	1.09 (0.97; 1.22)	**0.01**
	Female	1.00 (ref)	0.90 (0.74; 1.08)	0.97 (0.81; 1.15)	0.91 (0.74; 1.10)	
Model 2	Male	1.00 (ref)	1.06 (0.94; 1.18)	1.07 (0.96; 1.19)	1.09 (0.96; 1.21)	**0.01**

Female	1.00 (ref)	0.90 (0.74; 1.08)	0.97 (0.81; 1.15)	0.91 (0.74; 1.11)

Model 1: adjusted for age, as well as for the quartiles of the most representative variable of the other clusters, respectively.
Model 2: adjusted for covariates of Model 1, plus education level, region of employment, work hours, and sleep duration.
*kilocalorie

Table S5. Cross-sectional associations between k-means four-cluster solution top hit diet exposure quartiles with cardiometabolic risk prevalence stratified by age (median split, $N_{\leq 41\ years} = 4133$, $N_{>41\ years} = 3957$). Values are presented as prevalence ratio (95% confidence interval). Significant p-values ($p < 0.05$) are marked in bold.

		Quartile 1	Quartile 2	Quartile 3	Quartile 4	$P_{interaction}$
**Cluster 1: Saturated Fat Intake (%kcal*)**						
Prevalent Cases / N	≤41 years	165 / 751	262 / 1090	280 / 1150	292 / 1142	
	>41 years	313 / 692	440 / 964	542 / 1083	618 / 1218	
Model 1	≤41 years	1.00 (ref)	1.09 (0.91; 1.29)	1.06 (0.88; 1.27)	1.07 (0.87; 1.29)	0.54
	>41 years	1.00 (ref)	0.95 (0.84; 1.07)	1.02 (0.90; 1.13)	1.00 (0.88; 1.12)	
Model 2	≤41 years	1.00 (ref)	1.09 (0.91; 1.30)	1.06 (0.88; 1.27)	1.06 (0.86; 1.28)	0.53
	>41 years	1.00 (ref)	0.95 (0.84; 1.07)	1.01 (0.90; 1.13)	0.99 (0.87; 1.11)	
**Cluster 2: Carbohydrate Intake (%kcal)**						
		**Quartile 1**	**Quartile 2**	**Quartile 3**	**Quartile 4**	$P_{interaction}$
Prevalent Cases / N	≤41 years	237 / 844	204 / 894	241 / 1001	317 / 1394	
	>41 years	614 / 1111	464 / 910	424 / 917	411 / 1019	
Model 1	≤41 years	1.00 (ref)	0.87 (0.73; 1.03)	0.97 (0.82; 1.14)	1.01 (0.84; 1.20)	**0.001**
	>41 years	1.00 (ref)	0.98 (0.89; 1.06)	0.92 (0.83; 1.01)	0.85 (0.75; 0.95)	
Model 2	≤41 years	1.00 (ref)	0.87 (0.72; 1.03)	0.97 (0.81; 1.14)	0.99 (0.82; 1.18)	**<0.001**
	>41 years	1.00 (ref)	0.98 (0.89; 1.06)	0.91 (0.82; 1.00)	0.85 (0.75; 0.94)	
**Cluster 2: Fiber Intake (g/1000kcal)**						
		**Quartile 1**	**Quartile 2**	**Quartile 3**	**Quartile 4**	$P_{interaction}$
Prevalent Cases / N	≤41 years	300 / 1131	287 / 1082	214 / 969	198 / 951	
	>41 years	506 / 889	470 / 942	474 / 1050	463 / 1076	
Model 1	≤41 years	1.00 (ref)	0.98 (0.85; 1.13)	0.81 (0.68; 0.96)	0.77 (0.63; 0.92)	0.17
	>41 years	1.00 (ref)	0.88 (0.80; 0.97)	0.82 (0.74; 0.91)	0.79 (0.70; 0.88)	
Model 2	≤41 years	1.00 (ref)	1.00 (0.86; 1.15)	0.83 (0.69; 0.98)	0.79 (0.65; 0.95)	0.15
	>41 years	1.00 (ref)	0.89 (0.80; 0.97)	0.83 (0.74; 0.91)	0.80 (0.71; 0.89)	
**Cluster 3: Eating Occasions / day**						
		**Quartile 1**	**Quartile 2**	**Quartile 3**	**Quartile 4**	$P_{interaction}$
Prevalent Cases / N	≤41 years	274 / 1032	288 / 1159	251 / 997	186 / 945	
	>41 years	375 / 715	531 / 991	504 / 1034	503 / 1217	
Model 1	≤41 years	1.00 (ref)	0.91 (0.78; 1.06)	0.91 (0.77; 1.07)	0.70 (0.57; 0.85)	0.64
	>41 years	1.00 (ref)	1.03 (0.93; 1.13)	0.96 (0.86; 1.06)	0.83 (0.73; 0.93)	
Model 2	≤41 years	1.00 (ref)	0.91 (0.78; 1.06)	0.92 (0.78; 1.09)	0.72 (0.58; 0.87)	0.62
	>41 years	1.00 (ref)	1.03 (0.93; 1.12)	0.96 (0.86; 1.06)	0.82 (0.72; 0.93)	
**Cluster 3: Energy Intake (kcal)**						
		**Quartile 1**	**Quartile 2**	**Quartile 3**	**Quartile 4**	$P_{interaction}$
Prevalent Cases / N	≤41 years	258 / 1119	247 / 1050	240 / 962	254 / 1002	
	>41 years	420 / 904	469 / 972	523 / 1060	501 / 1021	
Model 1	≤41 years	1.00 (ref)	0.93 (0.78; 1.09)	0.91 (0.75; 1.09)	0.89 (0.72; 1.09)	0.79
	>41 years	1.00 (ref)	0.99 (0.88; 1.09)	0.99 (0.88; 1.10)	0.99 (0.87; 1.12)	
Model 2	≤41 years	1.00 (ref)	0.93 (0.78; 1.10)	0.92 (0.76; 1.10)	0.91 (0.73; 1.11)	0.82
	>41 years	1.00 (ref)	0.99 (0.89; 1.10)	0.99 (0.88; 1.11)	1.00 (0.88; 1.13)	
**Cluster 4: Protein Intake (%kcal)**						
		**Quartile 1**	**Quartile 2**	**Quartile 3**	**Quartile 4**	$P_{interaction}$
Prevalent Cases / N	≤41 years	179 / 764	283 / 1106	252 / 1046	285 / 1217	
	>41 years	258 / 580	488 / 1038	568 / 1116	599 / 1223	
Model 1	≤41 years	1.00 (ref)	1.08 (0.91; 1.27)	1.00 (0.84; 1.19)	0.97 (0.80; 1.16)	0.34
	>41 years	1.00 (ref)	1.06 (0.94; 1.18)	1.12 (1.00; 1.24)	1.07 (0.94; 1.20)	
Model 2	≤41 years	1.00 (ref)	1.08 (0.91; 1.27)	1.00 (0.84; 1.19)	0.97 (0.80; 1.16)	0.32
	>41 years	1.00 (ref)	1.06 (0.94; 1.18)	1.12 (1.00; 1.24)	1.07 (0.95; 1.20)	
**Cluster 4: Carbohydrate Irregularity (%)**						
		**Quartile 1**	**Quartile 2**	**Quartile 3**	**Quartile 4**	$P_{interaction}$
Prevalent Cases / N	≤41 years	208 / 878	226 / 919	308 / 1304	257 / 1032	
	>41 years	365 / 828	395 / 863	611 / 1216	542 / 1050	
Model 1	≤41 years	1.00 (ref)	1.00 (0.84; 1.18)	0.97; 0.82; 1.13)	0.99 (0.82; 1.18)	0.16
	>41 years	1.00 (ref)	1.01 (0.90; 1.13)	1.09 (0.98; 1.19)	1.06 (0.94; 1.17)	

Model 2	≤41 years	1.00 (ref)	1.00 (0.84; 1.18)	0.97 (0.82; 1.14)	0.98 (0.82; 1.17)	0.17
	>41 years	1.00 (ref)	1.01 (0.90; 1.12)	1.08 (0.98; 1.19)	1.05 (0.93; 1.17)	

Model 1: adjusted for age and sex, as well as for the quartiles of the most representative variable of the other clusters, respectively.
Model 2: adjusted for covariates of Model 1, plus education level, region of employment, work hours, and sleep duration.
*kilocalorie

Table S6. Cross-sectional associations between k-means four-cluster solution top hit diet exposure quartiles with cardiometabolic risk prevalence stratified by physical activity ($N_{low\ activity} = 7154$, $N_{high\ activity} = 936$). Values are presented as prevalence ratio (95% confidence interval). Significant p-values ($p < 0.05$) are marked in bold.

		Quartile 1	Quartile 2	Quartile 3	Quartile 4	$P_{interaction}$
**Cluster 1: Saturated Fat Intake (%kcal*)**						
Prevalent Cases / N	Low activity	415 / 1258	625 / 1820	726 / 1969	813 / 2107	
	High activity	63 / 185	77 / 234	96 / 264	97 / 253	
Model 1	Low activity	1.00 (ref)	1.00 (0.90; 1.12)	1.03 (0.92; 1.15)	1.02 (0.90; 1.14)	0.63
	High activity	1.00 (ref)	0.90 (0.65; 1.20)	0.96 (0.70; 1.27)	0.94 (0.66; 1.27)	
Model 2	Low activity	1.00 (ref)	1.00 (0.90; 1.12)	1.03 (0.92; 1.15)	1.01 (0.90; 1.14)	0.57
	High activity	1.00 (ref)	0.90 (0.65; 1.21)	0.97 (0.71; 1.29)	0.94 (0.66; 1.27)	
**Cluster 2: Carbohydrate Intake (%kcal)**						
		**Quartile 1**	**Quartile 2**	**Quartile 3**	**Quartile 4**	$P_{interaction}$
Prevalent Cases / N	Low activity	758 / 1736	595 / 1614	588 / 1715	638 / 2089	
	High activity	93 / 219	73 / 190	77 / 203	90 / 324	
Model 1	Low activity	1.00 (ref)	0.93 (0.85; 1.02)	0.92 (0.83; 1.01)	0.91 (0.82; 1.01)	0.36
	High activity	1.00 (ref)	0.94 (0.70; 1.19)	0.98 (0.74; 1.24)	0.77 (0.55; 1.04)	
Model 2	Low activity	1.00 (ref)	0.93 (0.85; 1.02)	0.91 (0.83; 1.00)	0.91 (0.81; 1.00)	0.32
	High activity	1.00 (ref)	0.93 (0.69; 1.19)	0.98 (0.73; 1.24)	0.77 (0.54; 1.03)	
**Cluster 2: Fiber Intake (g/1000kcal)**						
		**Quartile 1**	**Quartile 2**	**Quartile 3**	**Quartile 4**	$P_{interaction}$
Prevalent Cases / N	Low activity	725 / 1796	669 / 1790	606 / 1778	579 / 1790	
	High activity	81 / 224	88 / 234	82 / 241	82 / 237	
Model 1	Low activity	1.00 (ref)	0.91 (0.83; 0.99)	0.81 (0.73; 0.89)	0.75 (0.67; 0.84)	0.63
	High activity	1.00 (ref)	0.96 (0.72; 1.24)	0.82 (0.59; 1.10)	0.85 (0.59; 1.16)	
Model 2	Low activity	1.00 (ref)	0.92 (0.83; 1.00)	0.82 (0.74; 0.91)	0.77 (0.68; 0.86)	0.55
	High activity	1.00 (ref)	0.95 (0.71; 1.23)	0.83 (0.60; 1.11)	0.88 (0.61; 1.20)	
**Cluster 3: Eating Occasions / day**						
		**Quartile 1**	**Quartile 2**	**Quartile 3**	**Quartile 4**	$P_{interaction}$
Prevalent Cases / N	Low activity	567 / 1526	724 / 1913	676 / 1796	612 / 1919	
	High activity	82 / 221	95 / 237	79 / 235	77 / 243	
Model 1	Low activity	1.00 (ref)	0.97 (0.88; 1.07)	0.94 (0.84; 1.04)	0.75 (0.66; 0.84)	0.46
	High activity	1.00 (ref)	1.10 (0.84; 1.38)	0.92 (0.67; 1.20)	0.83 (0.59; 1.13)	
Model 2	Low activity	1.00 (ref)	0.97 (0.88; 1.07)	0.94 (0.85; 1.05)	0.75 (0.66; 0.85)	0.38
	High activity	1.00 (ref)	1.09 (0.83; 1.37)	0.91 (0.67; 1.19)	0.83 (0.59; 1.13)	
**Cluster 3: Energy Intake (kcal)**						
		**Quartile 1**	**Quartile 2**	**Quartile 3**	**Quartile 4**	$P_{interaction}$
Prevalent Cases / N	Low activity	588 / 1754	631 / 1801	686 / 1824	674 / 1775	
	High activity	90 / 269	85 / 221	77 / 198	81 / 248	
Model 1	Low activity	1.00 (ref)	0.96 (0.86; 1.06)	0.96 (0.85; 1.07)	0.97 (0.85; 1.09)	0.38
	High activity	1.00 (ref)	0.96 (0.70; 1.27)	0.91 (0.64; 1.24)	0.79 (0.53; 1.12)	
Model 2	Low activity	1.00 (ref)	0.97 (0.87; 1.07)	0.97 (0.86; 1.08)	0.98 (0.87; 1.11)	0.31
	High activity	1.00 (ref)	0.97 (0.70; 1.28)	0.92 (0.64; 1.25)	0.80 (0.53; 1.13)	
**Cluster 4: Protein Intake (%kcal)**						
		**Quartile 1**	**Quartile 2**	**Quartile 3**	**Quartile 4**	$P_{interaction}$
Prevalent Cases / N	Low activity	395 / 1197	696 / 1913	719 / 1905	769 / 2139	
	High activity	42 / 147	75 / 231	101 / 257	115 / 301	
Model 1	Low activity	1.00 (ref)	1.07 (0.96; 1.18)	1.06 (0.95; 1.18)	1.01 (0.90; 1.13)	0.24
	High activity	1.00 (ref)	1.05 (0.73; 1.45)	1.21 (0.86; 1.63)	1.15 (0.79; 1.57)	
Model 2	Low activity	1.00 (ref)	1.07 (0.96; 1.18)	1.06 (0.95; 1.18)	1.01 (0.90; 1.13)	0.21
	High activity	1.00 (ref)	1.04 (0.72; 1.44)	1.21 (0.85; 1.63)	1.15 (0.80; 1.58)	
**Cluster 4: Carbohydrate Irregularity (%)**						
		**Quartile 1**	**Quartile 2**	**Quartile 3**	**Quartile 4**	$P_{interaction}$
Prevalent Cases / N	Low activity	500 / 1492	555 / 1583	815 / 2226	709 / 1853	
	High activity	73 / 214	66 / 199	104 / 294	90 / 229	
Model 1	Low activity	1.00 (ref)	1.02 (0.92; 1.13)	1.05 (0.95; 1.15)	1.04 (0.93; 1.15)	0.54
	High activity	1.00 (ref)	0.92 (0.66; 1.22)	1.04 (0.78; 1.33)	1.02 (0.74; 1.34)	

Model 2	Low activity	1.00 (ref)	1.02 (0.92; 1.13)	1.05 (0.95; 1.15)	1.03 (0.93; 1.15)	0.48
	High activity	1.00 (ref)	0.91 (0.66; 1.22)	1.03 (0.78; 1.33)	1.00 (0.72; 1.32)	

Model 1: adjusted for age and sex, as well as for the quartiles of the most representative variable of the other clusters, respectively.
Model 2: adjusted for covariates of Model 1, plus education level, region of employment, work hours, and sleep duration.
*kilocalorie

Table S7. Cross-sectional associations between k-means four-cluster solution top hit diet exposure quartiles with cardiometabolic risk prevalence within overweight individuals (body mass index [BMI] ≥ 25 kg/m², *N* = 5416). Values are presented as prevalence ratio (95% confidence interval). Significant p-values ($p < 0.05$) are marked in bold.

	**Cluster 1: Saturated Fat Intake (%kcal*)**				
	**Quartile 1**	**Quartile 2**	**Quartile 3**	**Quartile 4**	P_{trend}
Prevalent Cases / N	442 / 980	644 / 1371	760 / 1515	831 / 1550	
Model 1	1.00 (ref)	1.01 (0.91; 1.12)	1.06 (0.95; 1.16)	1.07 (0.96; 1.19)	0.21
Model 2	1.00 (ref)	1.01 (0.90; 1.11)	1.05 (0.94; 1.16)	1.06 (0.95; 1.18)	0.28
	**Cluster 2: Carbohydrate Intake (%kcal)**				
	**Quartile 1**	**Quartile 2**	**Quartile 3**	**Quartile 4**	P_{trend}
Prevalent Cases / N	794 / 1435	623 / 1250	612 / 1272	648 / 1459	
Model 1	1.00 (ref)	0.93 (0.85; 1.01)	0.94 (0.86; 1.02)	0.92 (0.83; 1.01)	0.10
Model 2	1.00 (ref)	0.92 (0.84; 1.00)	0.94 (0.85; 1.02)	0.92 (0.83; 1.01)	0.08
	**Cluster 2: Fiber Intake (g/1000kcal)**				
	**Quartile 1**	**Quartile 2**	**Quartile 3**	**Quartile 4**	P_{trend}
Prevalent Cases / N	744 / 1415	699 / 1378	632 / 1341	602 / 1282	
Model 1	1.00 (ref)	0.97 (0.89; 1.05)	0.88 (0.79; 0.96)	0.89 (0.80; 0.99)	**0.009**
Model 2	1.00 (ref)	0.97 (0.89; 1.05)	0.88 (0.80; 0.97)	0.90 (0.81; 0.99)	**0.02**
	**Cluster 3: Eating Occasions / day**				
	**Quartile 1**	**Quartile 2**	**Quartile 3**	**Quartile 4**	P_{trend}
Prevalent Cases / N	603 / 1234	757 / 1456	691 / 1372	626 / 1354	
Model 1	1.00 (ref)	1.06 (0.97; 1.15)	1.01 (0.92; 1.11)	0.89 (0.79; 0.99)	**0.01**
Model 2	1.00 (ref)	1.06 (0.97; 1.15)	1.01 (0.92; 1.11)	0.89 (0.79; 0.99)	**0.009**
	**Cluster 3: Energy Intake (kcal)**				
	**Quartile 1**	**Quartile 2**	**Quartile 3**	**Quartile 4**	P_{trend}
Prevalent Cases / N	603 / 1243	660 / 1296	706 / 1429	708 / 1448	
Model 1	1.00 (ref)	0.99 (0.90; 1.09)	0.94 (0.84; 1.04)	0.97 (0.86; 1.09)	0.66
Model 2	1.00 (ref)	1.00 (0.90; 1.09)	0.94 (0.85; 1.05)	0.98 (0.87; 1.09)	0.76
	**Cluster 4: Protein Intake (%kcal)**				
	**Quartile 1**	**Quartile 2**	**Quartile 3**	**Quartile 4**	P_{trend}
Prevalent Cases / N	383 / 800	700 / 1389	770 / 1486	824 / 1741	
Model 1	1.00 (ref)	1.00 (0.90; 1.11)	0.99 (0.89; 1.09)	0.88 (0.78; 0.99)	**0.004**
Model 2	1.00 (ref)	1.01 (0.90; 1.11)	0.99 (0.89; 1.10)	0.88 (0.78; 0.99)	**0.005**
	**Cluster 4: Carbohydrate Irregularity (%)**				
	**Quartile 1**	**Quartile 2**	**Quartile 3**	**Quartile 4**	P_{trend}
Prevalent Cases / N	519 / 1079	558 / 1139	848 / 1698	752 / 1500	
Model 1	1.00 (ref)	1.01 (0.91; 1.11)	0.99 (0.90; 1.08)	0.97 (0.87; 1.06)	0.40
Model 2	1.00 (ref)	1.01 (0.91; 1.11)	0.99 (0.90; 1.08)	0.96 (0.87; 1.06)	0.38

Model 1: adjusted for age, sex, and BMI, as well as for the quartiles of the most representative variable of the other clusters, respectively.

Model 2: adjusted for covariates of Model 1, plus education level, region of employment, work hours, and sleep duration.

*kilocalorie

Table S8. Cross-sectional associations between k-means four-cluster solution top hit diet exposure quartiles with impaired blood glucose control prevalence (*N* = 8090). Values are presented as prevalence ratio (95% confidence interval). Significant p-values ($p < 0.05$) are marked in bold.

Cluster 1: Saturated Fat Intake (%kcal*)					
	**Quartile 1**	**Quartile 2**	**Quartile 3**	**Quartile 4**	P_{trend}
Prevalent Cases / N	542 / 1443	840 / 2054	942 / 2233	1047 / 2360	
Model 1	1.00 (ref)	1.11 (1.02; 1.20)	1.13 (1.05; 1.24)	1.19 (1.08; 1.29)	**<0.001**
Model 2	1.00 (ref)	1.11 (1.01; 1.20)	1.14 (1.04; 1.24)	1.17 (1.07; 1.28)	**<0.001**
**Cluster 2: Carbohydrate Intake (%kcal)**					
	**Quartile 1**	**Quartile 2**	**Quartile 3**	**Quartile 4**	P_{trend}
Prevalent Cases / N	820 / 1955	735 / 1804	833 / 1918	983 / 2413	
Model 1	1.00 (ref)	1.01 (0.93; 1.09)	1.10 (1.02; 1.19)	1.08 (0.99; 1.17)	**0.02**
Model 2	1.00 (ref)	1.01 (0.93; 1.09)	1.10 (1.01; 1.18)	1.08 (0.99; 1.17)	**0.04**
**Cluster 2: Fiber Intake (g/1000kcal)**					
	**Quartile 1**	**Quartile 2**	**Quartile 3**	**Quartile 4**	P_{trend}
Prevalent Cases / N	833 / 2020	844 / 2024	819 / 2019	875 / 2027	
Model 1	1.00 (ref)	0.96 (0.89; 1.04)	0.90 (0.82; 0.98)	0.94 (0.86; 1.02)	0.09
Model 2	1.00 (ref)	0.97 (0.89; 1.04)	0.91 (0.83; 0.99)	0.95 (0.87; 1.04)	0.17
**Cluster 3: Eating Occasions / day**					
	**Quartile 1**	**Quartile 2**	**Quartile 3**	**Quartile 4**	P_{trend}
Prevalent Cases / N	692 / 1747	875 / 2150	884 / 2031	920 / 2162	
Model 1	1.00 (ref)	0.98 (0.90; 1.07)	1.01 (0.93; 1.10)	0.94 (0.86; 1.03)	0.21
Model 2	1.00 (ref)	0.98 (0.90; 1.06)	1.01 (0.92; 1.10)	0.93 (0.84; 1.02)	0.13
**Cluster 3: Energy Intake (kcal)**					
	**Quartile 1**	**Quartile 2**	**Quartile 3**	**Quartile 4**	P_{trend}
Prevalent Cases / N	880 / 2023	841 / 2022	859 / 2022	791 / 2023	
Model 1	1.00 (ref)	0.97 (0.89; 1.04)	1.02 (0.93; 1.10)	0.99 (0.90; 1.08)	0.94
Model 2	1.00 (ref)	0.97 (0.90; 1.05)	1.02 (0.94; 1.11)	1.00 (0.90; 1.09)	0.82
**Cluster 4: Protein Intake (%kcal)**					
	**Quartile 1**	**Quartile 2**	**Quartile 3**	**Quartile 4**	P_{trend}
Prevalent Cases / N	570 / 1344	880 / 2144	891 / 2162	1030 / 2440	
Model 1	1.00 (ref)	0.97 (0.89; 1.06)	0.98 (0.89; 1.06)	1.04 (0.95; 1.13)	0.21
Model 2	1.00 (ref)	0.98 (0.89; 1.06)	0.98 (0.90; 1.07)	1.04 (0.95; 1.13)	0.17
**Cluster 4: Carbohydrate Irregularity (%)**					
	**Quartile 1**	**Quartile 2**	**Quartile 3**	**Quartile 4**	P_{trend}
Prevalent Cases / N	730 / 1706	769 / 1782	1040 / 2,520	832 / 2082	
Model 1	1.00 (ref)	1.00 (0.92; 1.08)	0.97 (0.90; 1.05)	0.95 (0.87; 1.03)	0.21
Model 2	1.00 (ref)	1.00 (0.92; 1.08)	0.97 (0.90; 1.05)	0.95 (0.87; 1.03)	0.18

Model 1: adjusted for age and sex, as well as for the quartiles of the most representative variable of the other clusters, respectively.
Model 2: adjusted for covariates of Model 1, plus education level, region of employment, work hours, and sleep duration.
*kilocalorie
